# The Lateral Preoptic Area: A Novel Regulator of Reward Seeking and Neuronal Activity in the Ventral Tegmental Area

**DOI:** 10.3389/fnins.2019.01433

**Published:** 2020-01-17

**Authors:** Adam G. Gordon-Fennell, Ryan G. Will, Vorani Ramachandra, Lydia Gordon-Fennell, Juan M. Dominguez, Daniel S. Zahm, Michela Marinelli

**Affiliations:** ^1^Department of Neuroscience, College of Natural Sciences, The University of Texas at Austin, Austin, TX, United States; ^2^Department of Psychology, College of Liberal Arts, The University of Texas at Austin, Austin, TX, United States; ^3^Division of Pharmacology and Toxicology, College of Pharmacy, The University of Texas at Austin, Austin, TX, United States; ^4^Department of Pharmacology and Physiology, School of Medicine, Saint Louis University, St. Louis, MO, United States; ^5^Department of Psychiatry, Dell Medical School, The University of Texas at Austin, Austin, TX, United States

**Keywords:** dopamine, punishment, cocaine, sucrose, reward, relapse, self-administration

## Abstract

The lateral preoptic area (LPO) is a hypothalamic region whose function has been largely unexplored. Its direct and indirect projections to the ventral tegmental area (VTA) suggest that the LPO could modulate the activity of the VTA and the reward-related behaviors that the VTA underlies. We examined the role of the LPO on reward taking and seeking using operant self-administration of cocaine or sucrose. Rats were trained to self-administer cocaine or sucrose and then subjected to extinction, whereby responding was no longer reinforced. We tested if stimulating the LPO pharmacologically with bicuculline or chemogenetically with Designer Receptors Exclusively Activated by Designer Drugs (DREADDs) modifies self-administration and/or seeking. In another set of experiments, we tested if manipulating the LPO influences cocaine self-administration during and after punishment. To examine the functional connectivity between the LPO and VTA, we used *in vivo* electrophysiology recordings in anesthetized rats. We tested if stimulating the LPO modifies the activity of GABA and dopamine neurons of the VTA. We found that stimulating the LPO reinstated cocaine and sucrose seeking behavior but had no effect on reward intake. Furthermore, both stimulating and inhibiting the LPO prevented the sustained reduction in cocaine intake seen after punishment. Finally, stimulating the LPO inhibited the activity of VTA GABA neurons while enhancing that of VTA dopamine neurons. These findings indicate that the LPO has the capacity to drive reward seeking, modulate sustained reductions in self-administration following punishment, and regulate the activity of VTA neurons. Taken together, these findings implicate the LPO as a previously overlooked member of the reward circuit.

## Introduction

The lateral preoptic area (LPO) is an anterior hypothalamic brain region whose function has been largely unexplored. Most studies have focused on its role in sleep and thirst (Osaka et al., 1993; Saad et al., 1996; Szymusiak et al., 2007). A small number of studies suggest that the LPO participates in reward behavior. Activating the LPO elicits locomotion (Shreve and Uretsky, 1989, 1991; Zahm et al., 2014; Lavezzi et al., 2015; Subramanian et al., 2018; Reichard et al., 2019a, b) and conditioned place preference (Reichard et al., 2019a). The LPO also supports intracranial electrical self-stimulation (Elder and Work, 1965; Bushnik et al., 2000), and neuronal activity in this structure is sensitive to fluctuations in cocaine levels during self-administration (Barker et al., 2015). The notion that the LPO might be important in reward is also supported by anatomical studies. The LPO sends monosynaptic glutamatergic and GABAergic projections to the ventral tegmental area (VTA) (Phillipson, 1979; Geisler and Zahm, 2005; Watabe-Uchida et al., 2012; Ogawa et al., 2014; Beier et al., 2015; Kalló et al., 2015), which is a critical regulator of drug seeking and taking. Stimuli that increase the activity of dopamine neurons of the VTA increase cocaine seeking and those that increase GABA transmission in the VTA decrease seeking (Phillips et al., 2003; Marinelli et al., 2006; Wise, 2013; Jin et al., 2018). Furthermore, the LPO projects to brain regions that influence the activity of VTA neurons, including, but not limited to, the lateral habenula (Mok and Mogenson, 1972; Kowski et al., 2008; Yetnikoff et al., 2015; Barker et al., 2017), rostromedial tegmental nucleus (Jhou et al., 2009; Yetnikoff et al., 2015), and dorsal raphe (Peyron et al., 1998; Ogawa et al., 2014). Taken together, these studies led us to postulate that the LPO modulates VTA activity and reward behavior.

In this paper, we examined the role of the LPO in reward behavior using operant self-administration of cocaine or sucrose. We measured the effects of stimulating the LPO on both self-administration and seeking. Self-administration tests are a direct measure of reward intake. In seeking tests, rats first learn to self-administer a reward, and then, they are subjected to an extinction phase, in which responding no longer delivers the reward. Seeking behavior can then be reinstated by different triggers to model “relapse” (Bossert et al., 2005). Here, we examined if stimulating the LPO produced reinstatement of seeking behavior. To increase external validity, we stimulated the LPO using two methods, pharmacologically by locally administering bicuculline (an antagonist of GABA-A receptors and calcium-activated potassium channels) and chemogenetically with excitatory Designer Receptors Exclusively Activated by Designer Drugs (DREADDs). We also examined the role of the LPO during and after punished responding for cocaine by pharmacologically stimulating the LPO with bicuculline or inhibiting it with baclofen + muscimol (agonists of GABA-B and GABA-A receptors, respectively) when the punishment was applied. Punishment involves learning the association between a response and an aversive stimulus which can lead to lasting reductions in self-administration (Ahmed, 2011; Vanderschuren et al., 2017). Finally, we investigated whether the LPO is functionally connected to the VTA by stimulating the LPO while recording neuronal activity in the VTA of anesthetized rats.

## Materials and Methods

### Subjects

Male Sprague Dawley rats weighing 250–300 g obtained from Harlan were housed by two to three in Plexiglas cages lined with chip bedding (P.J. Murphy, Montville, NJ, United States, cat#: Sani-Chips) and given *ad libitum* access to water and laboratory chow (LabDiet, St. Louis, MO, United States, cat#: 5053). Rats were maintained on a 12-h reverse light–dark cycle, and experiments began 1–4 h into the dark cycle. Procedures were done in accordance with the National Institutes of Health Guide for the Care and Use of Laboratory Animals and were approved by the Institutional Animal Care and Use Committee of The University of Texas at Austin.

### Drugs and Viral Vectors

The following drugs were obtained from Henry Schein (Dublin, OH, United States): isoflurane (cat#: 1169567762), meloxicam (cat#: 6451603845), flunixin meglumine (cat#: 049622), carprofen (cat#: 1311749), 0.9% saline (cat#: 002477), sodium brevital (cat#: 038431), and cefazolin (cat#: 1026761). The following drugs were obtained from Sigma-Aldrich (St. Louis, MO, United States): phosphate-buffered saline (cat#: P3813), (±)-baclofen (cat#: B5399), muscimol hydrobromide (cat#: G019), sucrose (cat#: S7903), paraformaldehyde (cat#: 158127), dimethyl sulfoxide (DMSO, cat#: D8418), and fast-green (cat#: F7252). (−)-Bicuculline methiodide (Bic, cat#: 2503) was obtained from Tocris (Bristol, United Kingdom). Artificial cerebral spinal fluid (aCSF, cat#: 59-7316) was obtained from Harvard Apparatus (Holliston, MA, United States). Betadine (cat#: 67618-155-32) was obtained from Purdue Products L.P. (Stamford, CT, United States). Clozapine-*n*-oxide (CNO) was supplied by the NIDA Drug Supply Program.

The following adeno-associated viral vectors were obtained from UNC Viral Vector Core to use in the chemogenetics (DREADDs) studies: rAAV5/hSyn-HA-hM3D(Gq)-IRES- mCitrine (hM3Dq; titer: 4e12 vg/mL), rAAV5/hSyn-ChR2 (E123A)-eYFP-WPRE (ChR2; titer: 3.4e12 vg/mL), and rAAV5/hSyn-eGFP (GFP; titer: 3.6e12 vg/mL).

Drugs injected intracranially were dissolved in aCSF. Drugs injected systemically were dissolved in sterile 0.9% saline. CNO was dissolved in DMSO before being diluted in saline (final concentration of DMSO 0.5%) or aCSF (final concentration of DMSO 0.0014%).

### Surgical Procedures

#### Anesthesia

For all surgical and *in vivo* electrophysiology procedures, anesthesia was induced by placing rats into an induction chamber (E-Z Anesthesia, Palmer, PA, United States) filled with 5% isoflurane regulated by an isoflurane vaporizer (E-Z Anesthesia, Palmer, PA, United States). Following induction, anesthesia was maintained with 2.0–2.5% isoflurane delivered via nose cone or stereotaxic breather (E-Z Anesthesia, Palmer, PA, United States). To ensure sufficient anesthesia, breathing rate, pinch response, and body temperature were monitored throughout procedures, and anesthesia was adjusted when necessary.

#### Intravenous Catheterization

Areas around incisions were shaved with electric clippers (Andis Company, Sturtevant, WI, United States, cat#: 22350), and the skin was cleaned with 10% betadine and sprayed with Lanacaine, which contains benzocaine (20%), benzethonium chloride (0.2%), and ethanol (36%). Intravenous silastic catheters were implanted in the right external jugular vein and passed under the skin to exit in the mid-scapular region. The catheters were accessible through a backport pedestal mount that was secured under the skin with surgical staples (Braintree Scientific, Inc., Braintree, MA, United States, cat#: ACS APL, EZC CS).

#### Intracranial Implantation of Guide-Cannulae and Viral Injection

Surgery sites were shaved the rat’s head was mounted in a stereotaxic apparatus (David Kopf Instruments, Tujunga, CA, United States, cat#: 902) with the upper body resting on a heating pad set at ∼37°C (Kent Scientific, Torrington, CT, United States). The local anesthetic mepivacaine (2%) was injected beneath the incision site, and the site was cleaned with 10% betadine. A scalpel incision was made, the tissue overlying the skull was removed, and a burr hole was drilled over the LPO. For experiments involving microinjections, a guide cannula (23-gauge thin-wall hypodermic tubing, 15-mm length Plastics One, Roanoke, VA, United States) was lowered at 18° to a site 2-mm above the LPO [final coordinate: AP: −0.12 mm, ML: −1.4 mm, DV: −8.6 mm from bregma, according to the Paxinos and Watson (2007) atlas]. The cannula was then fixed to the skull with skull screws and dental cement (Coltène/Whaledent Inc., Cuyahoga Falls, OH, United States, cat#: H00325). For experiments involving viral injections, a custom-made stainless steel injection cannula (30-gauge, BD Precisionglide, Franklin Lakes, NJ, United States, cat#: 305128) coupled to a gas-tight 5 nL Hamilton syringe and micropump (Harvard Apparatus, Holliston, MA, United States, cat#: Pump11) was lowered at 18° into the LPO (final coordinate: AP: −0.12 mm, ML: −1.4 mm, DV: −8.6 mm from bregma). A total of 500 nL was injected unilaterally at a rate of 100 nL/min over 5 min, and the cannula was left in place for 5 min before slowly retracting. At the conclusion of the surgery, the scalp was closed using surgical staples (Braintree Scientific, Inc., Braintree, MA, United States). Catheterization and intracranial surgeries were performed serially.

#### Surgical Recovery

At the conclusion of each surgery, wounds were covered with topical antibiotic ointment (Medique Products, Fort Myers, FL, United States, cat#: 22373). Systemic NSAID analgesics, either meloxicam (2.5 mg/kg/2 mL, s.c.), carprofen (5 mg/kg/mL, s.c.), or flunixin meglumine (2.5 mg/kg/0.5 mL, s.c.), were administered the day of surgery and 1 day following. Systemic antibiotic cefazolin (50 mg/kg/0.5 mL, i.v.) was administered the day of surgery and 2–6 days following (except for three rats in Experiment 1, which did not receive antibiotic).

Following implantations of guide-cannulae, rats were allowed to recover for at least 10 days prior to starting self-administration. Following surgeries involving viral injections, rats were allowed to recover for at least 6 days before starting self-administration, and we allowed at least 6 weeks of incubation prior to activating the DREADDs with CNO, to ensure adequate expression of DREADDs.

### Self-Administration

#### Acquisition of Self-Administration

All self-administration procedures took place in Med Associates chambers (Med Associates, Fairfax, VT, United States, cat#: CT-ENV-007-VP-X) outfitted with three horizontal photo-beam sensors to track locomotion (Med Associates, Fairfax, VT, United States, cat#: ENV-253SD) and two nose-holes (Med Associates, Fairfax, VT, United States, cat#: ENV-114BM) to track responding (nose-poking). Nose-poking into one hole (“active hole”) concomitantly delivered a reinforcer and a 10-s light cue within the hole. The onset of reinforcement coincided with the onset of time-out. Nose-poking into the other hole (“inactive hole”) had no consequences and was used to track non-goal-directed nose-poking. We recorded number of nose pokes, beam breaks, and reinforcements delivered using MED-PC IV (Med Associates, Fairfax, VT, United States).

For cocaine self-administration, the rat’s backport was connected to a Tygon tubing (Cole Parmer, Vernon Hills, IL, United States, cat#: 06419-01) coupled to an infusion pump (Med Associates, Fairfax, VT, United States, cat#:PHM108), which allowed delivery of cocaine (600 μg/kg/100 μL, i.v.). For sucrose self-administration, sucrose pellets (45 mg, Bioserv, Flemington, NJ, United States, cat#: F06233) were delivered through a pellet dispenser and receptacle (Med Associates, Fairfax, VT, United States, cat#: ENV-203M-45 and ENV-200R2M, respectively), located between the nose-holes. Cocaine and sucrose self-administration sessions were 90 min long and were conducted daily for 7–15 days, according to the experiment. Time-outs were of 10 s for the first 10 or 20 reinforcers, 20 s for the next ten, and 30 s from then onward, to prevent overdosing during cocaine self-administration. In a subset of experiments, we tested the effect of stimulating the LPO on self-administration of sucrose of cocaine, by pharmacologically stimulating the LPO immediately prior to placing rats in the operant chamber on the last day of self-administration. For all rats self-administering cocaine, at the conclusion of the self-administration procedure, and prior to starting the extinction procedure, we tested catheter patency by administering the fast acting anesthetic sodium brevital (5 mg/kg/0.5 mL, i.v.). Rats not immediately anesthetized were eliminated from the study.

#### Extinction

Following self-administration, responding was extinguished by running rats through identical conditions as the self-administration procedure but without delivering the primary reinforcer (cocaine or sucrose). The cue light in the active hole continued to be delivered with the same schedule as self-administration. The last day of extinction, we tested the effect of stimulating the LPO on cocaine or sucrose seeking by stimulating the LPO immediately prior to (pharmacological stimulation) or immediately upon (chemogenetic stimulation) placing rats in the operant chamber.

#### Punishment

During punishment, every reinforcer was punished with a coincident electric foot-shock (800 ms, 0.32–0.44 mA, mean = 0.36 mA) produced by a shock generator and administered through the operant chamber floor (Med Associates, Fairfax, VT, United States, cat#: ENV-414 and CT-ENV-OO5D+T, respectively). Shock amplitude was determined for each rat individually such that the shock produced flinching without producing freezing. To determine this amplitude, on the day prior to punishment, each rat received three to four test shocks starting with 0.3 mA and then of higher or lower intensities to titrate to their personal “flinching” response. During punishment, we tested the effect of manipulating the LPO on punished responding by pharmacologically stimulating or inhibiting the LPO immediately prior to placing rats in the operant chamber.

### Intracranial Microinjections

On the day prior to microinjections, we lowered a custom-made stainless steel dummy injection cannula (30-gauge) into the LPO (2 mm below the injector guide) for 30 s while loosely holding the rat. Microinjections were performed via custom-made stainless steel injection cannulae (30-gauge), connected to a micropump (Harvard Apparatus, Holliston, MA, United States, cat#: Pump11) via PE10 tubing. On the day of the microinjections, we lowered the injection cannula into the LPO, waited 30 s, injected 300 nL of drug over 60 s, and then waited 60 s to allow for diffusion, before removing the injector. Drugs were administered at the following concentrations, unilaterally into the LPO: bicuculline (80.4 ng base/300 nL) and baclofen + muscimol (64.1 ng/300 nL and 5.85 ng/300 nL, respectively). These doses were based on previous studies (Yetnikoff et al., 2015).

### Extracellular Electrophysiology Recordings of VTA Neurons

Rats were mounted in a stereotaxic apparatus (David Kopf Instruments, Tujunga, CA, United States, cat#: 902) and a local anesthetic (2% mepivacaine) was injected subcutaneously at the incision site before an incision was made. Burr holes were drilled in the skull at sites overlaying the LPO and VTA. A microinjection pipette was lowered into the LPO at a lateralward angle of 18° from vertical (to reach a final coordinate: AP: −0.12 mm, ML: −1.4 mm, DV: −8.6 mm from bregma). VTA recordings were performed with a glass pipette (WPI, Sarasota, FL, United States, cat# 1B150F-4) that was pulled with a vertical puller (Narishige, Amityville, NY, United States, cat#:PE-2), broken under a microscope to a tip diameter of 1–2 μm, and filled with 2% fast-green in a 2 M saline solution. The impedance of the glass pipette was 1.5–2.1 MOhms measured at 135 Hz (Winston Electronics, St. Louis, MO, United States, cat#:BL1000-B). The pipette was slowly lowered to the VTA (final coordinate: AP: −5.4 mm, ML: −0.6 mm, DV: −8.3 mm from bregma) with a hydraulic microdrive (David Kopf Instruments, Tujunga, CA, United States, cat#: 640). Extracellular voltage was amplified (Fintronics Inc., Orange, CT, United States), passed through a Hum Bug 50/60 Hz Noise Eliminator (Quest Scientific, North Vancouver, BC, Canada), and monitored on an oscilloscope (EZ Digital, Gwang-Ju City, South Korea, cat#: OS-5020A) and audio monitor (Grass Technologies, West Warwick, RI, United States, cat#: AM10). Signals were also digitized and recorded using AxoScope software [Molecular Devices, San Jose, CA, United States, cat#: Digidata 1440A (digitizer) and version: 10.7 (software)] running on a desktop computer. Neurons were classified as putative dopamine neurons based on established extracellular recording criteria: wide (>2.4 ms) waveform, measured from start to end when recorded with 400–500 Hz filters (Einhorn et al., 1988) and wide (>1.1 ms) waveform, measured from start to trough when recorded with 50–800 Hz filters (Ungless and Grace, 2012; Marinelli and McCutcheon, 2014), triphasic (+/−/+) waveform, and firing rate between 1 and 10 Hz. These criteria are ∼90% accurate at detecting neurons containing tyrosine hydroxylase (Ungless and Grace, 2012). We analyzed firing rate (spikes over time) and firing pattern. Dopamine neurons exhibit intermittent bursts, which are clusters of high-frequency spikes that start with an interspike interval of 80 ms and terminate with an interspike interval >160 ms (Grace and Bunney, 1983). The amount of bursting activity was calculated as the percentage of spikes emitted in bursts over the total number of spikes. We also calculated the frequency of burst events and the properties of the bursts (burst duration in ms). To determine the weight of bursting vs. non-bursting activity on overall firing rate, we analyzed “non-bursting activity” by subtracting burst events from the firing trace and by analyzing non-burst events separately. For this analysis, the spikes preceding and following each burst event were removed because their timing could be influenced by factors initiating and terminating burst events. Neurons were classified as putative GABA if they failed to meet the dopaminergic criteria. These neurons often have biphasic waveforms and comparatively high firing rates. We recorded baseline activity over a 3-min period, microinjected bicuculline (80.4 ng base/300 nL/3 min) or aCSF (300 nL/3 min) into the LPO over 3 min, and recorded for an additional 3 min after the end of microinjection. Only 1 neuron was recorded per rat to eliminate confounds stemming from multiple injections. At the conclusion of the recording, rats were euthanized and fast-green was ejected from the recording pipette into the end location by passing 28.6 mA cathodal current through the electrode with a current generator (Fintronics Inc., Orange, CT, United States, cat#: VL-1200 D). Neurons were excluded if any of the following criteria were met: (1) they were lost before 3 min post-microinjection; (2) the microinjector placement was outside the LPO; (3) the fast-green location was outside the VTA; and (4) neuronal activity had >12% baseline firing variability.

#### Validation of the Chemogenetic DREADD hM3Dq

To validate the activation of the excitatory DREADD hM3Dq-stimulated LPO neurons, we used a modified version of the recording procedures described above, in rats receiving a 5:3 cocktail of hM3Dq and ChR2 vectors. A burr hole was drilled over the LPO, and a triple barrel probe was lowered at an 18° angle to the LPO (final coordinate: AP: −0.12 mm, ML: −1.4 mm, DV: −8.6 mm from bregma). Neurons were recorded across multiple tracks in and around the LPO. The triple barrel was modeled based on previous studies (Mahler et al., 2014) and consisted of a recording pipette, as outline above, an injection pipette (ringcaps, Hirschmann, Eberstadt, Germany) pulled and broken-back at a ∼20 μm tip and positioned ∼100 μm behind the recording tip, and a 200 μm 0.39 NA optic fiber (Thorlabs, Newton, NJ, United States, cat#: FT200UMT) positioned ∼600 μm behind the recording tip (Supplementary Figure 2). This approach allowed us to identify ChR2 expressing neurons that have a high likelihood of co-expressing hM3Dq. For optic identification, we applied 473 nm laser stimulation (Laser Glow, Toronto, ON, Canada, cat#: LD-WL206) driven by a pulse train generator (Prizmatix, Israel, cat#: Pulser) at 0.2 Hz, 5 ms pulses, 2–20 mW. Neurons were classified as expressing ChR2 if they were excited upon laser stimulation (Cohen et al., 2012), with an average spike latency of <5 ms from pulse onset and an average jitter (standard deviation of spike latency) of <2 ms across 20 repeated stimulations. In a subset of neurons, we further verified ChR2 expression by also measuring fidelity (# spikes/# light pulses) at high frequency stimulation by delivering six 1 s-long trains (40 Hz, 5 ms pulses, 2–10 mW), at 9 s inter-train interval. Once a neuron was identified as expressing ChR2, we measured the effect of hM3Dq activation by locally injecting 30–60 nL of 10 μM CNO via pneumatic pulses (8–12 psi, 50–100 ms) delivered by a Picospritzer III (Parker, Cleveland, OH, United States) over 1–2 min. One to two neurons were recorded for each rat, with >30 min and >300 μm in-between injection sites, to minimize effects of CNO diffusion. At the conclusion of each experiment, fast-green was deposited and located as outlined below.

### Histology

The locations of recording sites, intracranial microinjection sites, and the distributions of the DREADD expression were determined at the conclusion of behavioral experiments. For electrophysiology experiments, rats were euthanized with isoflurane at the end of the recording. Brains were removed and fixed in 10% formalin for >24 h. For experiments involving microinjections, rats were euthanized with CO_2_ and brains were removed and stored in formalin for >24 h. For experiments involving DREADDs, rats were deeply anesthetized with isoflurane and transcardially perfused with Sorensen’s buffer (0.01 M PB, 2.5% sucrose, and 0.9% NaCl) followed by 4% paraformaldehyde in phosphate buffer solution (0.1 M PB, 2.5% sucrose, and 4% paraformaldehyde). Brains were then removed and post fixed in 4% paraformaldehyde in phosphate buffer solution for 24 h then transferred to 25% sucrose solution for ∼3 days until they were fully sunk. For all experiments, coronal brain sections were collected at 40 μm on a cryostat (Thermo Fisher Scientific, Waltham, MA, United States, cat# HM550) and then imaged with a microscope (Carl Zeiss, Oberkochen, Germany, cat#: Axio Zoom.V16). The recording site for electrophysiology was determined by locating and imaging the fast-green spot and then mapping it onto the corresponding section of the Paxinos and Watson (2007) atlas and a house made atlas that localized the VTA following immunohistochemistry for tyrosine hydroxylase. Following fast-green localization, the relative position of recorded neurons was back-calculated. The location of the microinjection sites was determined by imaging the ventral-most position of the injector track and then mapping it onto the corresponding section of the Paxinos and Watson atlas. The distribution of the chemogenetic constructs was determined by imaging brain sections with fluorescent microscopy and then mapping the distribution of the fluorescence on the corresponding section of the Paxinos and Watson atlas. Rats were removed from experiments when microinjections were located outside the LPO. The location of the misplaced microinjections and their corresponding behavioral data are shown in Supplementary Figure 6.

### Procedures

#### Experiment 1: Effects of Pharmacological Stimulation of the LPO on Cocaine Self-Administration and Seeking

Rats were allowed to self-administer cocaine for 90 min every day, for 7–8 days. The fixed-ratio requirement to obtain cocaine was 1 for all days (i.e., 1 nose poke: 1 infusion). Prior to the last day of self-administration, rats were assigned to the bicuculline (*n* = 6) or aCSF control (*n* = 9) groups in a way that minimized differences in infusions between groups. To test the effect of stimulating the LPO on cocaine taking, rats received an intra-LPO microinjection of bicuculline or aCSF on the last day of self-administration. Following cocaine self-administration, rats underwent extinction sessions for 90 min every day, for 19–20 days. To test the effect of stimulating the LPO on extinguished seeking behavior, rats received an intra-LPO microinjection of bicuculline or aCSF control on the last day of extinction (day 20 or 21). During the last 3 days of self-administration, one subject was identified as an outlier using Grubbs’ test extreme studentized deviate (ESD) method (subject mean of 263 compared to group mean of 46.47); thus, this subject was removed from the experiment. Primary statistical results were not affected by removing this subject.

#### Experiment 2: Effects of Chemogenetic Stimulation of the LPO on Cocaine Seeking

Rats expressing hM3Dq (*n* = 7) or GFP control (*n* = 9) in the LPO were allowed to self-administer cocaine for 90 min every day, for 10 days. The fixed-ratio requirement to obtain cocaine was 1 for days 1–3, 3 for days 4–6, and 5 for day 7 onward. Fixed ratios >1 were used to enhance discrimination between the active and inactive holes. Following cocaine self-administration, rats underwent extinction sessions for 90 min every day, for 21 days. To test the effect of stimulating the LPO on extinguished seeking behavior, rats received an intravenous injection of CNO (0.3 mg/kg/0.5 mL) on the last day of extinction.

#### Experiment 3: Effects of Pharmacological Stimulation of the LPO on Sucrose Self-Administration and Seeking

Rats were allowed to self-administer sucrose for 90 min every day, for 14–15 days. The fixed-ratio requirement and pellets per delivery (FR ratio–pellets per delivery) were FR1-1 for days 1–4, FR3-1 for day 5, FR5-1 for day 6, FR5-3 for days 7 and 8, and FR5-5 for days 9 and onward. One group of rats (*n* = 11) was started on FR1-5 for 2 days prior to FR1-1, but was changed to FR1-1 because rats were only eating a small proportion of the delivered pellets. There was no significant difference in behavior over the remaining self-administration days between rats started on FR1-5 and those that started on FR1-1, so the data were pooled and the first 2 days were excluded from analysis. Prior to the last day of self-administration, rats were assigned to bicuculline (*n* = 10) or aCSF control (*n* = 8) groups in a way that minimized differences in deliveries between groups. To test the effect of stimulating the LPO on sucrose taking, rats received the intra-LPO microinjection of bicuculline or aCSF on the last day of self-administration. Following sucrose self-administration, rats underwent extinction sessions for 90 min every day, for 26 days. To test the effect of stimulating the LPO on extinguished seeking behavior, rats received an intra-LPO microinjection of bicuculline or aCSF control on the last day of extinction.

#### Experiment 4: Effects of Pharmacological Manipulation of the LPO on Cocaine Self-Administration After Punishment

Rats were allowed to self-administer cocaine for 90 min every day, for 7 days. The fixed-ratio requirement for reward was 1 for days 1–4 and 3 for day 5 onward. Prior to undergoing punishment, rats were assigned to bicuculline (*n* = 6), baclofen + muscimol (*n* = 6), or aCSF control (*n* = 8) groups in a way that minimized differences in infusions between groups. To test the effects of LPO manipulation during and after punishment, rats received an intra-LPO microinjection of bicuculline, baclofen + muscimol, or aCSF on the day of punishment (day 8). We determined if punishment led to sustained changes in behavior by testing self-administration for 1 day of post-punishment (day 9).

#### Experiment 5: Effects of Pharmacological Stimulation of the LPO on the Activity of VTA Neurons

We recorded the activity of putative GABA and putative dopamine neurons in the VTA and measured their response to an intra-LPO microinjection of bicuculline (GABA: *n* = 8, dopamine: *n* = 9) or aCSF control (GABA: *n* = 6, dopamine: *n* = 7).

### Statistical Analysis and Data Visualization

In behavioral experiments, operant conditioning variables were analyzed using analysis of variance (ANOVA). Each variable was analyzed independently with group as a between-subject factor and experimental day as a within-subject factor. Additionally, responding was also analyzed using active hole and inactive hole as a within-subject factor. Tukey’s honest significant difference (HSD) was used for *post hoc* tests.

In electrophysiology experiments, the characteristics of neuron firing were expressed as delta from baseline (average of 3 min prior to the microinjection) and were analyzed with ANOVA. Each variable was analyzed independently with group and neuron type as between-subjects factors and time relative to microinjection (binned in 1 min intervals) as within-subjects factor, when relevant. HSD was used for *post hoc* tests.

For all experiments, *P* < 0.05 was used as a threshold for significance across statistical tests. All data are expressed as mean ± SEM. Sample sizes were calculated based on variance obtained from previous or preliminary experiments and on effect size (partial eta-squared = 0.01–0.25 for repeated measures or main effects ANOVA). Power was set at 0.80.

All statistical analysis was completed in R (version 3.5.0). ANOVA was computed using the “afex” package (version 0.21-2), HSD was computed using the “emmeans” package (version 1.2.3), and paired *t*-tests were computed using base R.

Data were visualized for publication using Graph Pad Prism (version 8.2.0). Images of brain placements (cannulae or viral expressions) were created in Adobe Illustrator CC (version 22.1) using the Paxinos and Watson digital atlas (Paxinos and Watson, 2007). All other figure aspects were created in Adobe Illustrator CC.

## Results

### Experiment 1: Pharmacological Stimulation of the LPO Promotes Cocaine Seeking, but Does Not Change Cocaine Self-Administration

**FIGURE 1:**
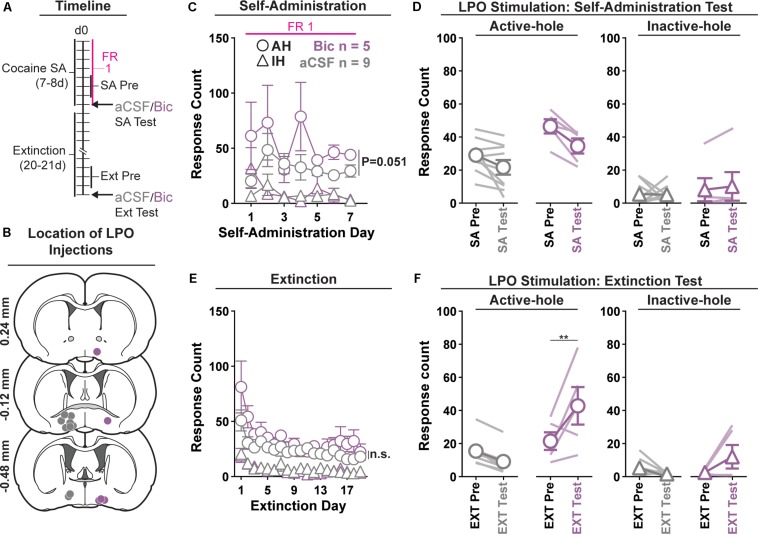
Pharmacological stimulation of the LPO promotes cocaine seeking, but does not change cocaine self-administration. **(A)** Timeline of behavioral procedures. SA, self-administration; FR: fixed ratio (number of responses required to obtain one cocaine infusion, depicted with pink line). **(B)** Location of LPO injections for aCSF (gray) and bicuculline (Bic, purple). **(C)** Cocaine self-administration behavior. There was slightly more responding in the active hole in rats that would later receive bicuculline compared with those that would later receive aCSF; however, during the last 3 days of self-administration that preceded the self-administration test, groups did not differ. **(D)** Self-administration test (SA test). Stimulating the LPO with bicuculline did not change active hole or inactive hole responding relative to aCSF or the average of the last 3 days of self-administration (SA Pre). **(E)** Extinction behavior. Both groups extinguished responding on the previously active hole. There was no difference between groups across extinction nor over the last 3 days of extinction (Ext Pre). **(F)** Extinction test (Ext Test). Stimulating the LPO with bicuculline reinstated cocaine seeking behavior, observed as increased responding on the previously active hole (HSD, ^∗∗^*P* < 0.01) but not inactive hole (HSD, *P* = 0.47). Symbols are means ± SEM for each group; lines are individual subjects. See main text for detailed statistics.

We determined if pharmacological stimulation of the LPO modulates cocaine self-administration or extinguished cocaine seeking behavior using operant conditioning (Figure 1).

#### Acquisition of Self-Administration

All rats acquired self-administration of cocaine (Figure 1C), as indicated by significant discrimination between the active hole and inactive hole (hole effect: *F*_(__1_,_12__)_ = 68.46, *P* < 0.001); however, this occurred differently across groups that would later receive intra-LPO microinjections of bicuculline or aCSF (group × hole interaction: *F*_(__1_,_12__)_ = 5.49, *P* = 0.037). These groups showed similar inactive hole responding, locomotion, and infusion counts (Supplementary Figure 1) (group effect: *F*_(__1_,_12__)_ = 0.71, 0.68, 0.86, *P* = 0.42, 0.42, 0.37, respectively). There was a trend toward more responding in the active hole in rats that would later receive bicuculline compared with those that would later receive aCSF (group effect: *F*_(__1_,_12__)_ = 4.68, *P* = 0.051) During the last 3 days of self-administration that preceded the self-administration test, groups did not differ in inactive hole responding, infusions counts, or locomotion (group effect: *F*_(__1_,_12__)_ = 0.20, 2.80, 0.62, *P* = 0.66, 0.12, 0.45, respectively). However, the group that would later receive intra-LPO microinjections of bicuculline had higher active hole responding compared with the group that would later receive intra-LPO aCSF (group effect: *F*_(__1_,_12__)_ = 9.91, *P* = 0.0084).

#### Self-Administration Test

During the self-administration test (Figure 1D), intra-LPO microinjections did not differentially modify responding relative to the last 3 days of self-administration (group × hole × day interaction: *F*_(__1_,_12__)_ = 0.67, *P* = 0.43) nor did they differentially modify infusion counts or locomotion (Supplementary Figure 1) (group × day interaction: *F*_(__1_,_12__)_ = 0.20, 0.39, *P* = 0.66, 0.54, respectively).

#### Extinction

Seeking, as measured by responding in the previously active hole, declined over the course of extinction sessions (Figure 1E), and this occurred similarly across groups (day effect: *F*_(__18_,_180__)_ = 9.18, *P* < 0.001; group × day interaction: *F*_(__18_,_180__)_ = 0.72, *P* = 0.79).

Groups did not differ over the last 3 days of extinction that preceded the extinction test, for active hole responding, inactive hole responding, or locomotion (group effect: *F*_(__1_,_12__)_ = 1.30, 1.31, 0.63, *P* = 0.28, 0.27, 0.44, respectively).

#### Extinction Test (Reinstatement)

During the extinction test, intra-LPO microinjections differentially modified responding (Figure 1F) (group × hole × day interaction: *F*_(__1_,_12__)_ = 9.98, *P* = 0.0082). Specifically, relative to the average of the last 3 days of extinction, bicuculline increased active hole responding (HSD, *P* = 0.0095), but aCSF did not (HSD, *P* = 0.67), and neither bicuculline nor aCSF modified inactive hole responding (HSD, Bic: *P* = 0.57; aCSF: *P* = 0.97). Additionally, bicuculline to increased locomotion (Supplementary Figure 1) (group × day interaction: *F*_(__1_,_12__)_ = 6.41, *P* = 0.026).

### Experiment 2: Chemogenetic Stimulation of the LPO Promotes Cocaine Seeking

**FIGURE 2:**
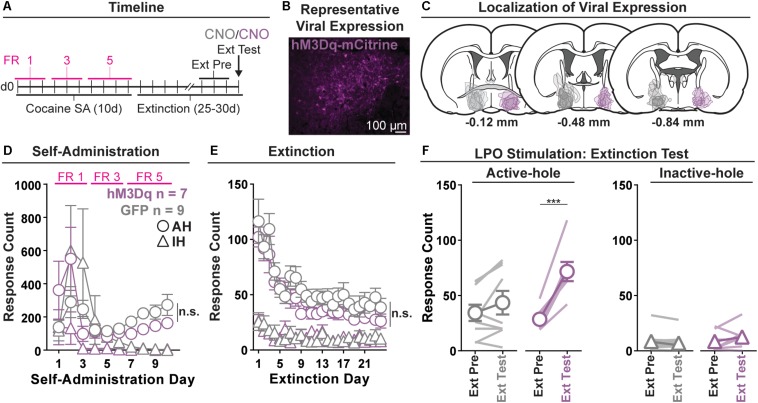
Chemogenetic stimulation of the LPO promotes cocaine seeking. **(A)** Timeline of behavioral procedures. SA, self-administration; FR, fixed ratio (number of responses required to obtain one cocaine infusion, depicted by pink lines). **(B)** Representative image of hM3Dq-mCitrine fluorescence in the LPO. **(C)** Localization of viral expression for GFP (gray) and hM3Dq (purple). **(D)** Cocaine self-administration behavior. Both groups acquired cocaine self-administration and there was no difference between groups across self-administration or over the last 3 days of self-administration (SA Pre). **(E)** Extinction behavior. Both groups extinguished responding on the previously active hole. There was no difference between groups across extinction or over the last 3 days of extinction (Ext Pre). **(F)** Extinction test (Ext Test). In the hM3Dq group, stimulating the LPO with CNO reinstated cocaine seeking behavior, observed as increased responding on the previously active hole (HSD, ^∗∗∗^*P* < 0.001) but not inactive hole (HSD, *P* = 0.99). Symbols are means ± SEM for each group; lines are individual subjects. See main text for detailed statistics.

We determined if chemogenetic stimulation of the LPO using hM3Dq modulates extinguished cocaine seeking behavior using operant conditioning (Figure 2) and validated the hM3Dq DREADD construct.

#### Validation of hM3Dq Stimulation

Neurons in the LPO were classified as co-expressing ChR2 and hM3Dq based on responses to optical stimulation. Low frequency stimulation (0.5 Hz, 10 ms pulses) of LPO neurons that co-expressed ChR2 and hM3Dq excited the neurons with short latency, low jitter, and high fidelity (Supplementary Figure 2A).

Local intra-LPO application of CNO to optically identified neurons increased firing in four out of six LPO neurons (Supplementary Figure 2), indicating that CNO stimulated neurons as intended.

#### Acquisition of Self-Administration

All rats acquired self-administration of cocaine (Figure 2D), as indicated by a significant discrimination between the active hole and inactive hole (hole effect: *F*_(__1_,_14__)_ = 5.36, *P* = 0.036), and this occurred similarly across rats in the hM3Dq and GFP groups (group × hole interaction: *F*_(__1_,_14__)_ = 2.14, *P* = 0.17). These groups also showed similar active hole responding, inactive hole responding, infusion counts, and locomotion (Supplementary Figure 3) (group: *F*_(__1_,_14__)_ = 0.07, 2.00, 1.12, 0.17, *P* = 0.80, 0.18, 0.31, 0.68, respectively).

#### Extinction

Seeking, as measured by responding in the previously active hole, declined over the course of extinction sessions (Figure 2E), and this occurred similarly across groups (day effect: *F*_(__23_,_332__)_ = 18.28, *P* < 0.001; group × day interaction: *F*_(__23_,_332__)_ = 0.56, *P* = 0.95).

Groups did not differ over the last 3 days of extinction that preceded the extinction test, for active hole responding, inactive hole responding, or locomotion (group effect: *F*_(__1_,_14__)_ = 0.40, 0.00, 0.60, *P* = 0.54, 0.99, 0.45, respectively).

#### Extinction Test (Reinstatement)

During the extinction test, administration of CNO differentially modified responding in the hM3Dq and GFP control groups (Figure 2F) (group × hole × day interaction: *F*_(__1_,_14__)_ = 15.21, *P* = 0.0016). Specifically, relative to the average of last 3 days of extinction, CNO increased active hole responding in the hM3Dq group (HSD, *P* < 0.001), but not in the GFP group (HSD, *P* = 0.35), and CNO did not modify inactive hole responding in either the hM3Dq or the GFP group (HSD, Bic: *P* = 0.99; aCSF: *P* = 1.00). Additionally, CNO had no differential effects on locomotion (Supplementary Figure 3) (group × day interaction: *F*_(__1,14__)_ = 2.46, *P* = 0.14).

### Experiment 3: Pharmacological Stimulation of the LPO Promotes Sucrose Seeking, but Does Not Change Sucrose Self-Administration

**FIGURE 3:**
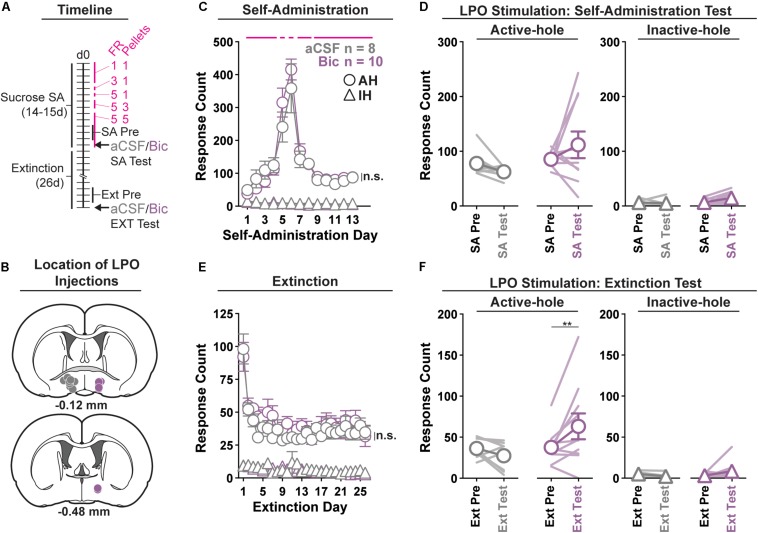
Pharmacological stimulation of the LPO promotes sucrose seeking, but does not change sucrose self-administration. **(A)** Timeline of behavioral procedures. SA, self-administration; FR, fixed ratio (number of responses required to obtain one reward delivery, depicted with pink lines). Pellets: number of pellets obtained per reward delivery, depicted with pink lines. **(B)** Location of LPO injections for aCSF (gray) and bicuculline (Bic, purple). **(C)** Sucrose self-administration behavior. Both groups acquired sucrose self-administration and there was no difference between groups across self-administration or over the last 3 days of self-administration (SA Pre). Rats updated responding with changes in FR schedule and number of rewards per delivery. **(D)** Self-administration test (SA Test). Stimulating the LPO with bicuculline did not change active hole or inactive hole responding relative to aCSF controls. **(E)** Extinction behavior. Both groups extinguished responding on the previously active hole. There was no difference between groups across extinction or over the last 3 days of extinction (Ext Pre). **(F)** Extinction test (Ext Test). Stimulating the LPO with bicuculline reinstated sucrose seeking behavior, observed as increased responding on the previously active hole (HSD, ^∗∗^*P* < 0.01) but not the inactive hole (HSD, *P* = 1.00). Symbols are mean ± SEM for each group; lines are individual subjects. See main text for detailed statistics.

In order to ascertain whether stimulation of the LPO has a general effect across rewards or is specific for cocaine, we repeated experiments with sucrose in place of cocaine. We determined if pharmacological stimulation of the LPO modulates sucrose self-administration or extinguished sucrose seeking behavior using operant conditioning (Figure 3).

#### Acquisition of Self-Administration

All rats acquired self-administration of sucrose (Figure 3C), as indicated by significant discrimination between the active hole and inactive hole (hole effect: *F*_(__1_,_16__)_ = 194.9, *P* < 0.001), and this occurred similarly in rats that would later receive intra-LPO microinjections of bicuculline or aCSF (group × hole interaction: *F*_(__1_,_16__)_ = 0.28, *P* = 0.60). These groups also showed similar active hole responding, inactive hole responding, number of pellets delivered, number of pellets eaten, and locomotion (Supplementary Figure 4) (group effect: *F*_(__1_,_16__)_ = 0.20, 0.42, 0.22, 0.20, 0.0040, *P* = 0.66, 0.53, 0.22, 0.66, 0.95, respectively).

During the last 3 days of self-administration that preceded the self-administration test, groups did not differ in active hole responding, inactive hole responding, number of pellets delivered, number of pellets eaten, nor locomotion (group effect: *F*_(__1_,_16__)_ = 0.73, 0.01, 1.25, 0.86, 0.0056, *P* = 0.41, 0.94, 0.28, 0.37, 0.52, respectively).

#### Self-Administration Test

During the self-administration test (Figure 3D), intra-LPO microinjections did not differentially modify responding relative to the last 3 days of self-administration (group × hole × day interaction: *F*_(__1_,_16__)_ = 1.31, *P* = 0.27) nor did they differentially modify number of pellets delivered, number of pellets eaten, nor locomotion (Supplementary Figure 4) (group × day interaction: *F*_(__1_,_16__)_ = 0.82, 0.89, 3.32, *P* = 0.38, 0.36, 0.087).

#### Extinction

Seeking, as measured by responding in the previously active hole, declined over the course of extinction sessions (Figure 3E), and this occurred similarly across groups (day effect: *F*_(__25_,_400__)_ = 12.85, *P* < 0.001; group × day interaction: *F*_(__25_,_400__)_ = 0.71, *P* = 0.85).

Groups did not differ over the last 3 days of extinction that preceded the extinction test, for active hole responding, inactive hole responding, or locomotion (group effect: *F*_(__1_,_16__)_ = 0.030, 2.73, 1.17, *P* = 0.87, 0.12, 0.30, respectively).

#### Extinction Test (Reinstatement)

During the extinction test, intra-LPO microinjections produced a trend to differentially modify responding (Figure 3F) (group × hole × day interaction: *F*_(__1_,_16__)_ = 4.12, *P* = 0.059). Specifically, relative to the average of last 3 days of extinction, bicuculline increased active hole responding (HSD, *P* = 0.0080), but aCSF did not (HSD, *P* = 0.92), and neither bicuculline nor aCSF modified inactive hole responding (HSD, Bic: *P* = 0.99; aCSF: *P* = 1.00). Additionally, bicuculline and aCSF had a differential effect on locomotion (Supplementary Figure 4) (group × day interaction: *F*_(__1_,_16__)_ = 6.81, *P* = 0.019). Specifically, relative to the average of last 3 days of extinction, bicuculline increased locomotion (HSD, *P* = 0.0024), but aCSF did not (HSD, *P* = 0.98).

### Experiment 4: Pharmacological Manipulation of the LPO Disrupts Reduction in Self-Administration of Cocaine After Punishment

**FIGURE 4:**
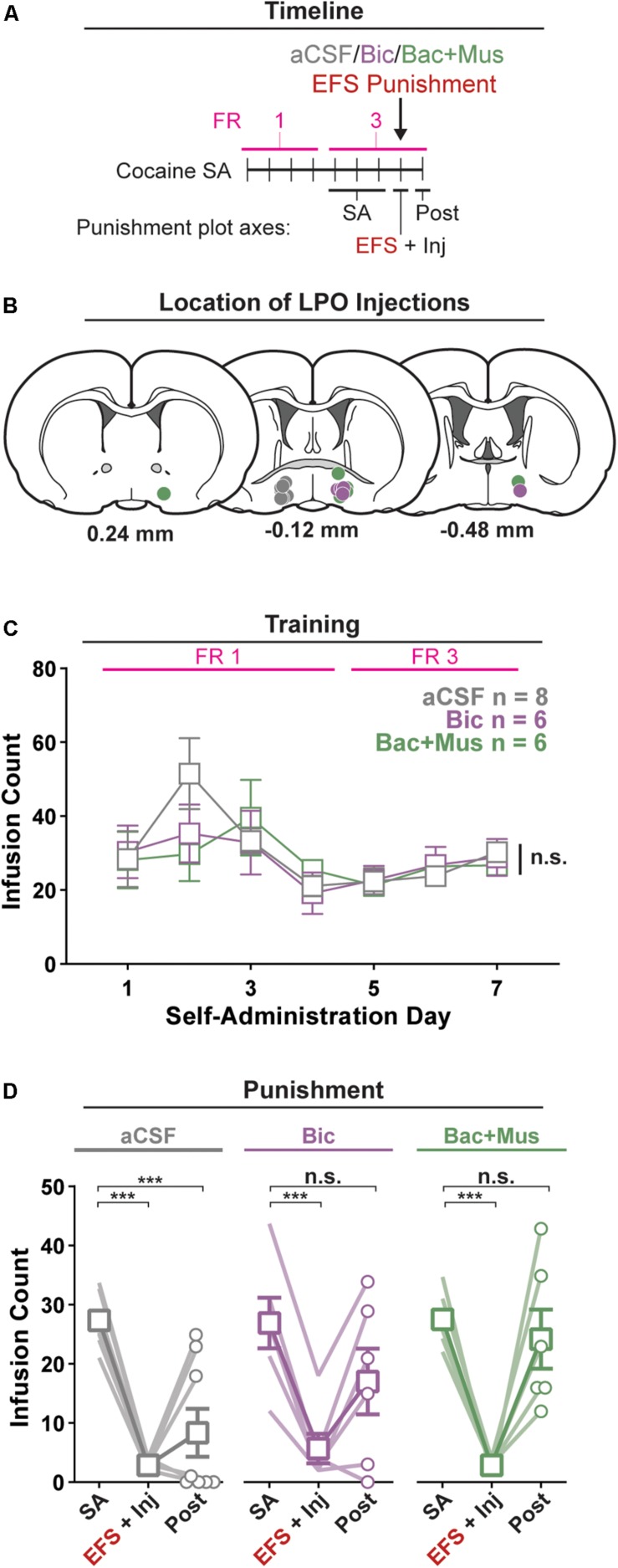
Pharmacological manipulation of the LPO disrupts the reduction in self-administration of cocaine after punishment. **(A)** Timeline of behavioral procedures. SA, self-administration; FR, fixed ratio (number of responses required to obtain one cocaine infusion, depicted with pink line). **(B)** Location of LPO injections for aCSF (gray), bicuculline (Bic, purple), and baclofen + muscimol (Bac + Mus, green). **(C)** Cocaine self-administration behavior, data are mean ± SEM of each group. There was no difference between groups across self-administration or over the last 3 days of self-administration (SA). **(D)** Behavior during punishment. Lines are individual subjects; symbols and error bars represent means ± SEM of each group. During footshock (EFS) punishment, all groups decreased the number of infusions relative to pre punishment (SA) (all HSD comparisons, *P*s < 0.001), and this occurred to a similar extent in animals receiving aCSF, bicuculline, or baclofen + muscimol. On the day following punishment (Post), only the aCSF group remained significantly below baseline intake (HSD, aCSF: ^∗∗∗^*P* < 0.001), whereas the other groups returned to pre-baseline intake (HSD, Bic: *P* = 0.20; Bac + Mus: *P* = 0.99).

We determined if pharmacological stimulation or inhibition of the LPO during punishment reduces cocaine self-administration during and after punishment, using operant conditioning (Figure 4).

#### Acquisition of Self-Administration

All rats acquired self-administration of cocaine, as indicated by a significant discrimination between the active hole and inactive hole (hole effect: *F*_(__1_,_17__)_ = 24.91, *P* < 0.001) (Supplementary Figure 5). This occurred similarly in groups that would later receive intra-LPO microinjection of aCSF, bicuculline, or baclofen + muscimol (group × hole interaction: *F*_(__2_,_17__)_ = 0.35, *P* = 0.71). These groups also showed similar active hole responding, inactive hole responding, infusion counts, and locomotion (Figure 4C and Supplementary Figure 5) (group effect: *F*_(__2_,_17__)_ = 0.35, 0.046, 0.19, 1.10, *P* = 0.71, 0.96, 0.83, 0.36, respectively).

Groups did not differ over the last 3 days of self-administration that preceded the punishment test, for active hole responding, or inactive hole responding, infusion counts, and locomotion (group effect: *F*_(__2_,_17__)_ = 0.086, 0.78, 0.013, 0.069, *P* = 0.92, 0.48, 0.99, 0.93, respectively).

#### Punishment

There was a significant difference in cocaine infusion counts across groups during the three phases of the procedure: average of the last 3 days of self-administration, electric footshock punishment, and post punishment (Figure 4D) (group × day interaction: *F*_(__4_,_34__)_ = 3.35, *P* = 0.020). Relative to the average of the last 3 days of self-administration, footshock punishment suppressed intake in all groups (all groups: HSD, *P*s < 0.001). However, rats that received aCSF showed a sustained decrease in cocaine infusions on the day following the punishment (HSD, *P* < 0.001), whereas rats that received bicuculline or baclofen + muscimol did not (HSD, *P* = 0.23, 0.99, respectively). Additionally, across the different phases of the procedure, groups did not differentially change active hole responding, inactive hole responding, or locomotion (Supplementary Figure 5) (group × day interaction, *F*_(__4_,_34__)_ = 1.89, 0.59, 1.64, *P* = 0.14, 0.67, 0.19).

### Experiment 5: Effects of Pharmacological Stimulation Enhances the Firing Rate of VTA Dopamine Neurons and Inhibits That of VTA GABA Neurons

**FIGURE 5:**
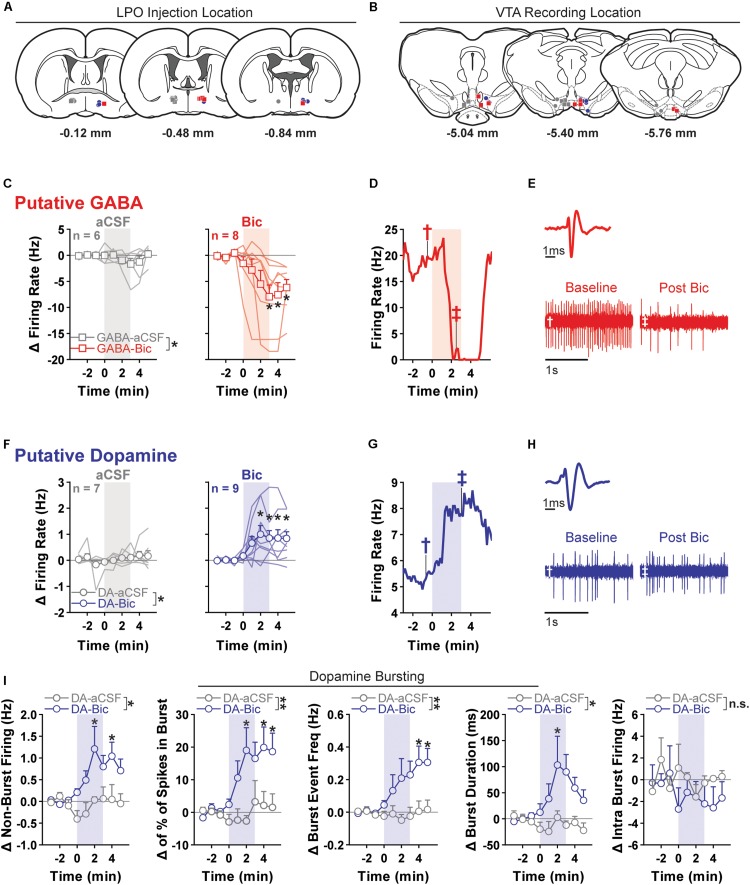
Pharmacological stimulation of the LPO enhances the firing rate of VTA dopamine neurons and inhibits that of VTA GABA neurons. **(A)** Location of LPO injections: aCSF (gray), bicuculline (Bic, red for GABA neurons and blue for dopamine neurons), during recordings of GABA neurons (squares) or dopamine neurons (circles). **(B)** Locations of dopamine (circles) and GABA (squares) neurons within the VTA. Color indicates corresponding intra-LPO injection: aCSF (gray), bicuculline (Bic, red for GABA neurons and blue for dopamine neurons). **(C)** Firing in GABA neurons (delta from baseline) before and after the administration of aCSF (gray) or bicuculline (Bic, red). Time is relative to onset of 3-min microinjection; each point represents the mean ± SEM values of each group. Stimulating the LPO with bicuculline decreased firing in GABA neurons relative to aCSF control and baseline (pre-injection) activity (group × time interaction: *F*_(__8_,_96__)_ = 3.29, *P* = 0.0023, HSD, ^∗^*P* < 0.05 compared with all pre-injection time-points). **(D)** Representative firing rate in a GABA neuron. There was substantial decrease in firing rate throughout injection and following. **(E)** Average waveform and recording traces for the neuron shown in graph **(D)**. Symbols denote the time period from which each trace was (obtained. **(F)** Firing in dopamine neuron (delta from baseline) before and after the administration of aCSF (gray) or bicuculline (Bic, blue). Time is relative to onset of the 3-min microinjection; each point represents the mean ± SEM values of each group. Stimulating the LPO with bicuculline increased the firing rate of dopamine neurons, relative to aCSF control and baseline (pre-injection) activity (group × time interaction: *F*_(__8_,_122__)_ = 2.87, *P* = 0.0060, HSD, ^∗^*P* < 0.05 compared with all pre-injection time-points). **(G)** Representative firing rate in a dopamine neuron. There was an increase in firing rate throughout the injection and following. **(H)** Average waveform and recording traces for the neuron shown in graph **(G)**. Symbols denote the time period from which each trace was obtained. **(I)** Burst characteristics of dopamine neurons before and after the administration of aCSF or Bic (delta from baseline) for non-burst frequency (Hz) [% of spikes emitted in bursts, burst event frequency (Hz), burst duration (ms), and intra burst frequency (Hz) (HSD, ^∗^*P* < 0.05 compared with all pre injection time-bins)]. Symbols are mean ± SEM for each group; lines are individual subjects. See main text for detailed statistics.)

We determined if pharmacological stimulation of the LPO modulates the activity of VTA neurons using *in vivo* anesthetized extracellular recordings (Figure 5).

Relative to aCSF control, stimulating the LPO with bicuculline had differential effects on putative GABA and dopamine neurons of the VTA (neuron type × group × time interaction: *F*_(__8_,_208__)_ = 4.62, *P* < 0.001) (Figures 5C,F).

In the case of putative GABA neurons, the average baseline firing rate (3 min preceding the microinjection) was 10.48 ± 1.48 Hz, and activity was similar in groups that would later receive intra-LPO microinjection of aCSF or bicuculline (group effect: *F*_(__1_,_12__)_ = 0.50, *P* = 0.49). Intra-LPO microinjection of bicuculline decreased firing relative to aCSF control and to baseline (Figure 5C) (group effect: *F*_(__1_,_12__)_ = 4.81, *P* = 0.049; group × time interaction: *F*_(__8_,_96__)_ = 3.29, *P* = 0.0023). The decrease in firing rate produced by bicuculline was significant during minutes 3, 4, and 5 after the start of the microinjection, compared with baseline (minutes -3, -2, and -1) (all comparisons: HSD, *P*s < 0.05). There were no significant changes in firing rate after aCSF at any time (all comparisons: HSD, *P*s > 0.98).

In the case of putative dopamine neurons, the average baseline firing rate (3 min preceding the microinjection) was 4.69 ± 0.69 Hz, and activity was similar in groups that would later receive intra-LPO microinjection of aCSF or bicuculline (group effect: *F*_(__1_,_14__)_ = 0.24, *P* = 0.63). Intra-LPO microinjection of bicuculline increased firing rate relative to aCSF control and to baseline (Figure 5F) (group effect: *F*_(__1_,_14__)_ = 5.82, *P* = 0.030; group × time interaction: *F*_(__8_,_112__)_ = 2.87, *P* = 0.0060). The increase in firing rate produced by bicuculline was significant during minutes 2, 3, 4, and 5 after the microinjection, compared with baseline (minutes -3, -2, and -1) (all comparisons: HSD, *P*s < 0.05). There were no significant changes in firing rate after aCSF at any time (all comparisons: HSD, *P*s > 0.98).

We also examined the firing pattern of dopamine neurons (Figure 5I). Relative to aCSF control, bicuculline increased non-burst firing rate (group × time interaction: *F*_(__8_,_112__)_ = 2.46, *P* = 0.017). This increase was significant during minutes 2 and 4 compared with baseline (minutes -3, -2, and -1) (all comparisons: HSD, *P* < 0.05). Bicuculline increased the amount of bursting measured as percent of spikes in bursts (group × time interaction: *F*_(__8_,_112__)_ = 3.06, *P* = 0.0037) and burst event frequency (group × time interaction: *F*_(__8_,_112__)_ = 3.12, *P* = 0.0032). The increase was significant during minutes 2, 4, and 5 for percent of spikes in bursts and minutes 4 and 5 for burst event frequency (all comparisons: HSD, *P* < 0.05). In those neurons that exhibited bursting activity (14/16), stimulation of the LPO produced a slight increase in burst duration (group × time interaction: *F*_(__8_,_96__)_ = 2.14, *P* = 0.039) that was significant during minute 2. There were no changes in intra-burst frequency (group × time interaction: *F*_(__8_,_96__)_ = 0.72, *P* = 0.67).

## Discussion

Our results indicate that stimulating the LPO precipitates reinstatement of reward seeking behavior for both cocaine and sucrose, but it does not alter cocaine or sucrose self-administration. Manipulating the LPO also prevents the reduction in cocaine self-administration after punishment. Finally, stimulating the LPO inhibits the activity of putative GABA neurons in the VTA and increases the activity of putative dopamine neurons.

### Stimulating the LPO Modulates Reward Behaviors

Previous studies showed that stimulating the LPO elicits conditioned place preference and locomotor activity. We therefore hypothesized that the LPO might play a role in reward (Reichard et al., 2019a). Here we studied it directly by measuring reward self-administration and seeking. Self-administration is a direct measure of reward intake. Seeking is measured by responding in the absence of the reward, and stimulus-induced increases in seeking (i.e., reinstatement of seeking behavior) are thought to model relapse (Bossert et al., 2005). Reinstatement of seeking behavior was observed after stimulating the LPO using two independent methods: pharmacology and chemogenetics. Pharmacological stimulation was achieved with bicuculline, an antagonist of GABA-A receptors and calcium-activated potassium channels, while chemogenetic stimulation was achieved with hM3Dq, a receptor that is coupled to an excitatory g-protein and stimulated by CNO. These convergent results provide higher confidence that stimulating the LPO precipitates reinstatement of cocaine seeking than either result alone. Pharmacological stimulation of the LPO precipitated reinstatement of seeking of both sucrose and cocaine, suggesting that the LPO serves a general function for reward seeking, rather than a specific function for cocaine seeking. In all cases, reinstatement of seeking led to selectively higher responding on the active compared with the inactive hole, indicating a specific enhancement of goal-directed seeking behavior, rather than simply a generalized increase in arousal or activity.

In contrast to findings that pharmacological stimulation of LPO increased seeking behavior, pharmacological stimulation of the LPO did not substantially increase sucrose or cocaine intake during self-administration. It is unlikely that this was due to a ceiling effect because on the day of LPO stimulation, intake and responding were lower than they were during the earlier phases of the self-administration procedure. These results showing that the LPO does not impact the consummatory aspect of rewards are consistent with previous findings showing that stimulation of the LPO does not modify consumption of food (Reichard et al., 2019a).

Drug intake during and after punishment have been used in self-administration studies to test the ability of punishment to act as a deterrent to future drug taking. Punishment, in the form of electric footshock, suppressed cocaine intake in all groups. Similar to what is reported in the literature, punishment was a deterrent for future drug intake in control rats, illustrated by intake levels remaining suppressed the day following punishment (Ahmed, 2011). However, this was not the case for rats that received either stimulation or inhibition of the LPO pharmacologically. These rats returned to baseline intake of cocaine the day after punishment, indicating that punishment was not a deterrent in these rats. These results suggest that normal activity patterns within the LPO during punishment are necessary to drive lasting changes in behavior following punishment. This effect was not explained by differences in the number of punishments received or the degree of suppression in cocaine intake, as all groups suppressed intake on the day of the punishment, and there were no differences in the number of punishments delivered. These results imply that the LPO is not only involved in reward seeking behaviors but also in long-term reductions in cocaine self-administration following punishment, without altering the acute effects of punishment. Previous studies showed that electric footshock, which is the punishment stimulus used here, enhances the activity of neurons within the LPO (Ono et al., 1986; Campeau and Watson, 1997; Martinez et al., 1998; Snowball et al., 2000; Briski and Gillen, 2001), but ours is the first to link activity in the LPO to sustained effects following punishment.

In our studies, we did not consistently observe an increase in locomotor activity after stimulating the LPO with bicuculline. This is in contrast to previous studies, which have consistently shown increases in locomotor activity in an open field (Shreve and Uretsky, 1989, 1991; Zahm et al., 2014; Lavezzi et al., 2015; Subramanian et al., 2018; Reichard et al., 2019a, b). One possible caveat is the method we used to measured locomotion in our studies. Our self-administration chambers allow changes in motor activity to be measured (Marinelli et al., 2003), but they might not be sensitive enough to detect the changes in locomotion that were observed with larger chambers equipped with more photo-beams. Another possibility is that in a passive context, such as an open field, stimulating the LPO may heighten exploration behavior, which manifests as an increase in locomotion. Instead, in an engaging context, such as self-administration, increased responding may compete with locomotion, wherein rats spent their time seeking reward, rather than moving throughout the chamber. The fact that stimulating the LPO triggered seeking is in line with the idea that stimulating the LPO could be driving fixed action patterns (Reichard et al., 2019a). In our case, stimulating the LPO after self-administration training and extinction may reengage fixed action patterns involved in self-administration.

Reinstatement of drug and food seeking behavior occurs after both rewarding and stressful stimuli (Venniro et al., 2016). Our data do not make clear if stimulating the LPO is mimicking rewarding or stressful stimuli to produce a reinstatement of seeking behavior. Reichard et al. (2019a) found that stimulating the LPO produces conditioned place preference. This suggests that stimulating the LPO may precipitate reinstatement by mimicking reward. However, additional studies will be needed to directly determine the valence of stimulating the LPO.

While our studies indicate that stimulating the LPO is sufficient to precipitate reinstatement of seeking, they do not indicate that neuronal activity within the LPO is necessary for reinstatement of seeking. Such studies would require inhibiting the LPO during drug, stress, or cue-precipitated reinstatement. Nevertheless, even if the activity in the LPO is not necessary for precipitated reinstatement, our results still indicate that the LPO is capable of driving the behavior.

### Stimulating the LPO Modulates VTA Neurons

The LPO projection to the VTA had long been described (Zahm et al., 2001; Colussi-Mas et al., 2007; Geisler et al., 2007; Watabe-Uchida et al., 2012; Beier et al., 2015; Kalló et al., 2015; Yetnikoff et al., 2015; Faget et al., 2016), but its functional connectivity had never been experimentally determined. Our results show that stimulating the LPO with bicuculline inhibits putative GABA neurons and stimulates putative dopamine neurons of the VTA. The inhibition of putative GABA neurons of the VTA was strong, some neurons completely stopped firing, only to slowly return to firing, while the excitation of putative dopamine neurons of the VTA was more modest. This excitation coincided with an increase in both non-bursting activity (the spikes emitted outside of burst events) and the amount of bursting (the percentage of spikes emitted in bursts, and frequency of burst events). The size of the bursts was slightly increased, but the frequency of the spikes within the bursts was not. This increase in neuronal activity is consistent with changes in synaptic input, specifically, an increase in glutamatergic input and a decrease in GABAergic input onto dopamine neurons (Paladini and Tepper, 1999; Lobb et al., 2010; Morikawa and Paladini, 2011).

While our study clearly indicates there is a functional connection between the LPO and subpopulations within the VTA, it does not reveal the mechanism by which the LPO regulates these subpopulations. One possibility is that LPO inhibition of VTA GABA neurons disinhibits VTA dopamine neurons. Our observation that stimulating the LPO leads to major suppression of GABA neurons and a slight enhancement of dopamine neurons is in line with this idea (Subramanian et al., 2018). However, the LPO also contains a mix of glutamate and GABA neurons (Kalló et al., 2015; Barker et al., 2017) that project to the VTA (Kalló et al., 2015). If both GABA and glutamate projections are functionally connected to both GABA and dopamine neurons in the VTA, then our results suggest that this functional connectivity is biased toward inhibition on GABA neurons and excitation on dopamine neurons, akin to what is observed in the lateral hypothalamus (Nieh et al., 2015). A final possibility is that our results reflect LPO connectivity with other intermediary structures. Indeed, the LPO sends projections to several other brain structures known to regulate the activity of VTA neurons (e.g., the lateral habenula, or rostromedial tegmental nucleus). Regardless of mechanism, detailed monosynaptic and poly-synaptic electrophysiological experiments will be necessary to definitively determine the nature of the functional connectivity.

We identified VTA neurons as putative GABA or dopamine based on established extracellular waveform and firing rate criteria (Ungless and Grace, 2012). We refer to these neuron populations as “putative” because we recognize the controversy around using extracellular criteria for identifying dopamine neurons in the VTA. However, using the extracellular identification technique we employed, there is high likelihood (88–93%) that neurons classified as dopamine would also be classified as such using immunohistochemistry (Ungless and Grace, 2012). Neurons that did not reach the criteria for classification as a dopamine neuron were classified as putative GABA neurons based on research indicating that GABA neurons are the second largest population of VTA neurons (∼35%) behind dopamine neurons (Nair-Roberts et al., 2008). We acknowledge that there may be glutamate neurons within the sample we identified as putative GABA neurons; however, glutamate neurons are a small portion of VTA neurons (∼2–3%) in the regions in which we recorded (Nair-Roberts et al., 2008).

### Connections Between the VTA and Reward Behaviors

Stimuli that increase the activity of dopamine neurons of the VTA trigger reinstatement of seeking behavior (Marinelli et al., 2006; Marinelli and McCutcheon, 2014). Similarly, dopamine receptor activation or increases in dopamine in VTA-projection areas such as the nucleus accumbens also precipitate reinstatement of cocaine seeking (De Vries et al., 1999; Schmidt et al., 2006). In addition, reducing the activity of dopamine neurons of the VTA or blocking dopamine receptors in the nucleus accumbens reduce cocaine seeking (Anderson et al., 2003, 2006; Bachtell et al., 2005; Marinelli et al., 2006; Xue et al., 2011). Therefore, the increase in activity of dopamine neurons we observed after LPO stimulation is a plausible mechanism underlying our findings, as shown for other behaviors (Zahm et al., 2014; Subramanian et al., 2018; Reichard et al., 2019a).

The role of GABA neurons of the VTA in reinstatement of drug seeking behaviors has not been extensively studied, but recent findings suggest that GABA neurons also play a role. Increasing GABA transmission in the VTA reduces dopamine levels in the nucleus accumbens and suppresses seeking behavior (Jin et al., 2018); it also attenuates the ability of cues to trigger reward seeking (Wakabayashi et al., 2019). Therefore, together, the decrease in activity of GABA neurons of the VTA and the increase in the activity of dopamine neurons could work to drive the reinstatement of seeking we observed. A similar regulation of behavior has been described in the lateral hypothalamus. Stimulation of lateral hypothalamus GABA neurons promotes behavioral activation (Barbano et al., 2016; Nieh et al., 2016; Tyree and de Lecea, 2017) through disinhibition of VTA dopamine (Nieh et al., 2016). This suggests that a functional connection from hypothalamic GABA neurons to GABA neurons of the VTA generalizes across the hypothalamus.

Changes in the activity of VTA neurons after manipulating the LPO could also be responsible for the observed effects on cocaine taking after punishment. The VTA exhibits heterogeneous responses after aversive stimuli (Volman et al., 2013). In a reward context, dopamine neurons can pause briefly in response to an aversive stimulus, such as the footshock punishment used here (McCutcheon et al., 2012; Holly and Miczek, 2016; Matsumoto et al., 2016), whereas GABA neurons increase activity (Tan et al., 2012). These temporally precise responses in the VTA have been proposed to be a “teaching signal” that allows making associations with stimuli (Schultz, 2007; Mileykovskiy and Morales, 2011; Tan et al., 2012; Creed et al., 2014; Stelly et al., 2019). Both stimulating and inhibiting VTA activity disrupts these temporally precise responses, and thereby prevents making associations with stimuli (Salinas-Hernandez et al., 2018). Similarly, in our studies, both stimulating and inhibiting the LPO was capable of disrupting sustained effects of punishment. It is possible that these manipulations, by disrupting the activity of VTA neurons, prevent the temporal changes in VTA activity and thus the association with punishment; this could be a possible mechanism underlying the effects seen after stimulation and inhibition of the LPO. At this point, this mechanism remains speculative.

## Conclusion

In conclusion, our results indicate that the LPO has the capacity to drive reward seeking, persistently reduce self-administration following punishment, and regulate the activity of VTA neurons. Taken together, the LPO may be a previously overlooked member of the reward circuit.

## Data Availability Statement

The raw data supporting the conclusions of this article will be made available by the authors, without undue reservation, to any qualified researcher.

## Ethics Statement

The animal study was reviewed and approved by the Institutional Animal Care and Use Committee of the University of Texas at Austin.

## Author Contributions

All authors listed have made a substantial, direct and intellectual contribution to the work, and approved it for publication.

## Conflict of Interest

The authors declare that the research was conducted in the absence of any commercial or financial relationships that could be construed as a potential conflict of interest.
