# Supplementary material for: The Lateral Preoptic Area: A Novel Regulator of Reward Seeking and Neuronal Activity in the Ventral Tegmental Area
Source: Front Neurosci. 2020 Jan 17;13:1433. doi: 10.3389/fnins.2019.01433 (PMC6978721; doi:10.3389/fnins.2019.01433)
Supplement: Supplementary file 1 [file Data_Sheet_1.pdf]

Supplementary figure 1: pharmacological stimulation of the LPO promotes cocaine seeking, but does not change cocaine self-administration

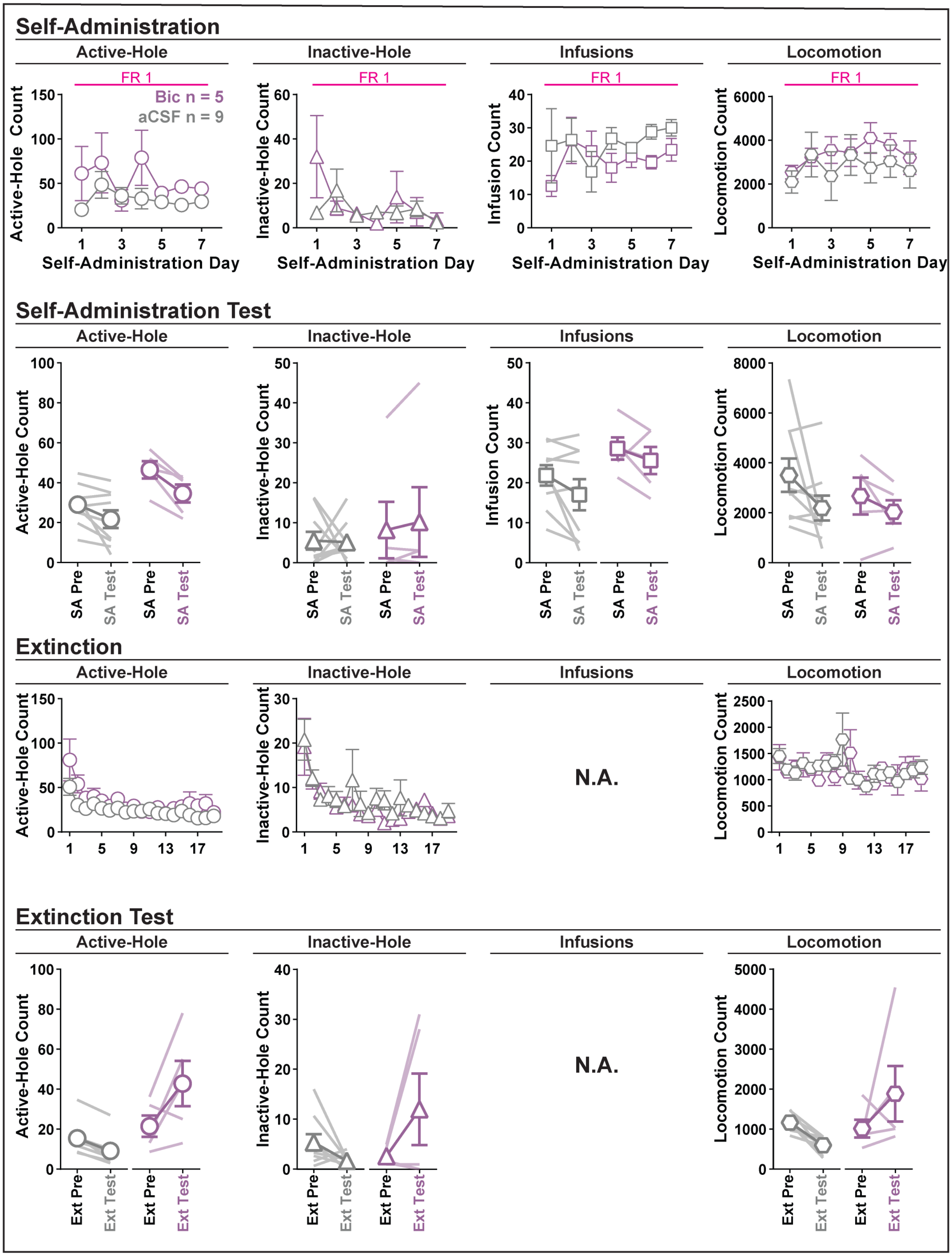

(1st row) Behavior during cocaine self-administration (SA). From left to right, active hole responding, inactive hole responding, infusions, and locomotion. **(2nd row)** Behavior during the self-administration test (SA Test). From left to right, active hole responding, inactive hole responding, infusions, and locomotion. Stimulating the LPO with bicuculline did not change active hole responding, inactive hole responding, infusions, or locomotion (group x day interaction: $F_{1,12}$, $P > 0.37$ for all comparisons) relative to aCSF or the average of the three last days of self-administration (SA Pre). **(3rd row)** Behavior during the extinction phase. From left to right: active hole responding, inactive hole responding, and locomotion. **(4th row)** Behavior during the extinction test (Ext Test). Stimulating the LPO with bicuculline increased active hole and inactive hole responding (group x day interaction: $F_{1,12} = 15.67, 6.35$, $P = 0.0019, 0.027$, respectively) relative to aCSF control and the average of the last three days of extinction (Ext Pre). There was trend for an increase in locomotion (group x day interaction $F_{1,12} = 6.41$, $P = 0.26$). Symbols are mean ± SEM for each group; lines are individual subjects. See main text for more detailed statistics.

Supplementary figure 2: Validation of the chemogenetic DREADD hM3Dq in LPO neurons

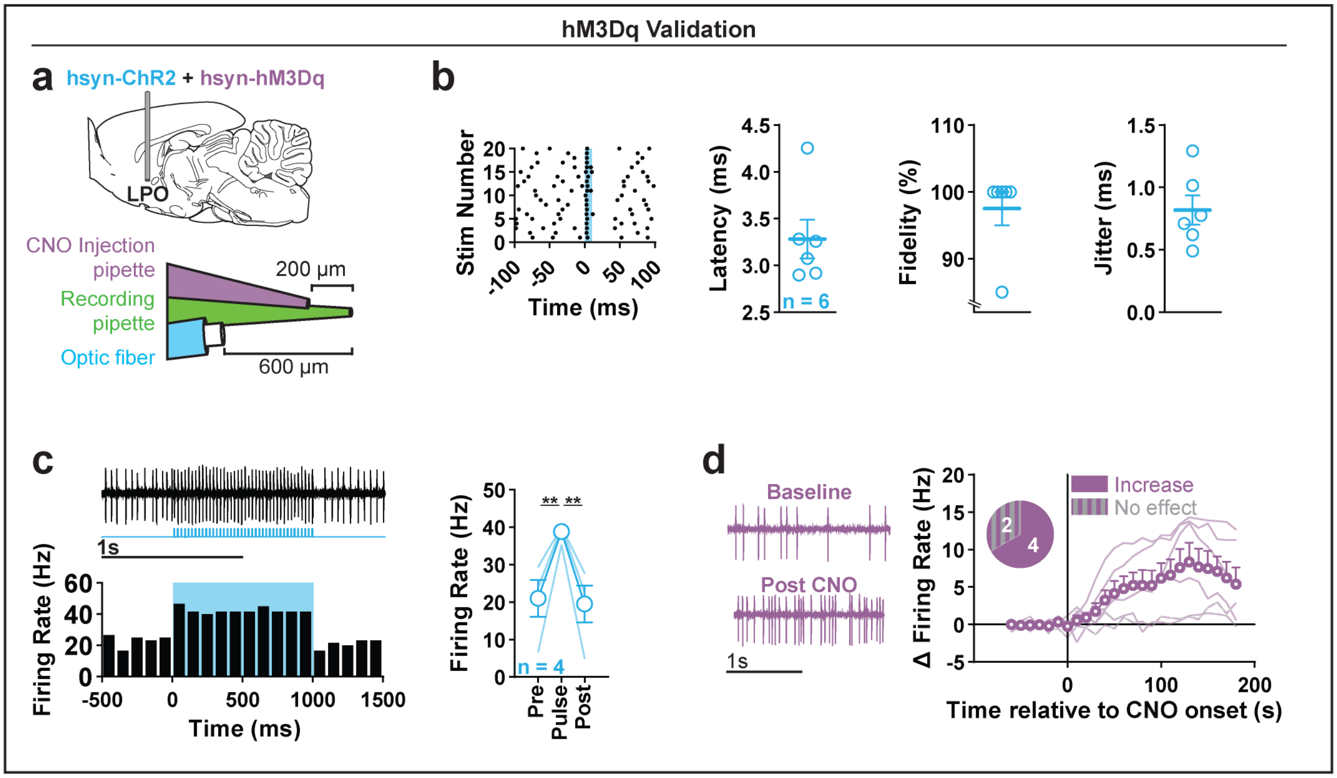

(a) Recording preparation (top), and diagram of the triple barrel pipette used for these experiments (bottom). **(b)** From left to right: peri-stimulus raster of firing in response to single pulse stimulations (0.2Hz, 10ms, 20 pulses), and latency, fidelity, and jitter to pulse stimulation. Horizontal lines are mean ± SEM and circles are individual neurons. **(c)** Representative trace of an LPO neuron (top) and average firing rate (bottom) in response to high-frequency stimulation (1s, 40Hz, 5ms pulse, 9s ITI, 6 trains, blue bars). Firing rate in responses to high frequency stimulation (right) (**HSD, P<0.01). Circles are mean ± SEM and lines are individual neurons. **(d)** Representative trace of an LPO neuron before (baseline) and after CNO application (Post CNO) (left) and firing rate relative to intra-LPO application of CNO (CNO onset) (right). Inset shows a pie chart of the number of neurons showing a change in firing or no change in firing. Circles are mean ± SEM and lines are individual neurons.

Supplementary figure 3: Chemogenetic stimulation of the LPO promotes cocaine seeking

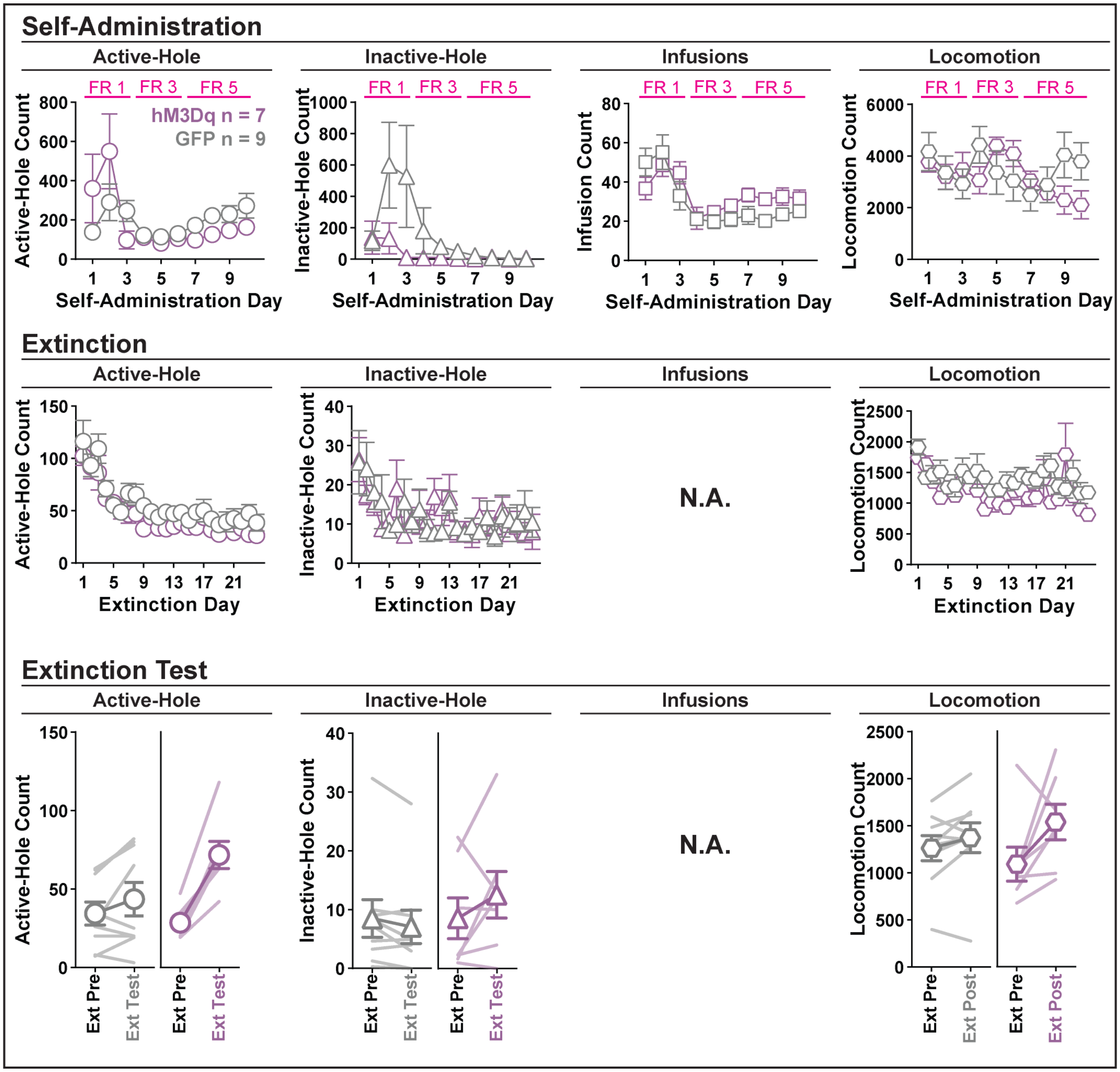

(1st row) Behavior during cocaine self-administration (SA); FR: fixed ratio (number of responses required to obtain one infusion of cocaine, depicted with pink lines). From left to right, active hole responding, inactive hole responding, infusions, and locomotion. **(2nd row).** Behavior during the extinction phase. From left to right: active hole responding, inactive hole responding, and locomotion. **(3rd row)** Behavior during the extinction test (Ext Test). Stimulating the LPO with hM3dq and CNO increased active hole, but not inactive hole responding (group x day interaction: $F_{1,14}$ = 17.05, 3.34, P < 0.001, P = 0.089, respectively) relative to aCSF control and the average of the last three days of extinction (Ext Pre). There was no effect on locomotion ($F_{1,14}$ = 2.46, P = 0.14). Symbols are mean ± SEM for each group; lines are individual subjects. See main text for more detailed statistics.

Supplementary figure 4: Pharmacological stimulation of the LPO promotes sucrose seeking but does not change sucrose self-administration

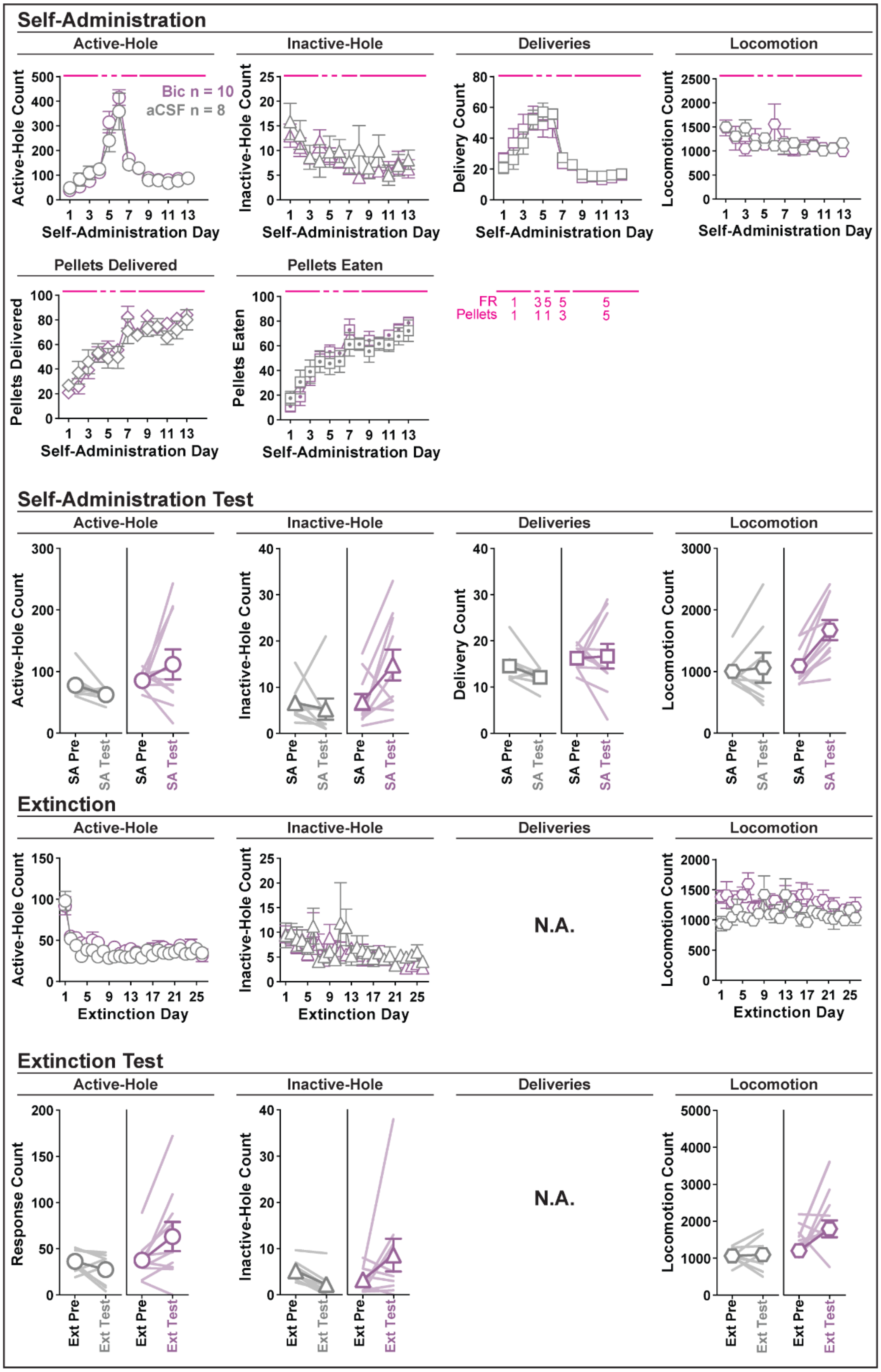

(1st row) Behavior during sucrose self-administration (SA); FR: fixed ratio (number of responses required to obtain reward, depicted with pink lines); Pellets: number of pellets obtained per reward delivery, (also depicted with pink lines). From left to right, active hole responding, inactive hole responding, delivery counts, pellets delivered, and pellets eaten. **(2nd row)** Behavior during the self-administration test (SA Test). Stimulating the LPO with bicuculline did not change active hole responding, delivery counts, or locomotion (group x day interaction: $F_{1,16}$, $P > 0.087$ for all comparisons) relative to aCSF control and the last three days of self-administration (SA Pre). Stimulating the LPO with bicuculline increased inactive hole responding ($F_{1,16} = 5.55$, $P = 0.032$). **(3rd row)** Behavior during the extinction phase. From left to right: active hole responding, inactive hole responding, and locomotion. **(4th row)** Behavior during the extinction test (Ext Test). Stimulating the LPO with bicuculline increased active hole, inactive hole responding, and locomotion (group x day interaction: $F_{1,16} = 6.91, 4.57, 6.81$, $P = 0.018, 0.048, 0.019$ respectively) relative to aCSF control and the average of the last three days of extinction (Ext Pre). Symbols are mean ± SEM for each group; lines are individual subjects. See main text for detailed statistics.

Supplementary figure 5: LPO pharmacological manipulation of the LPO disrupts the reduction in self-administration of cocaine after punishment

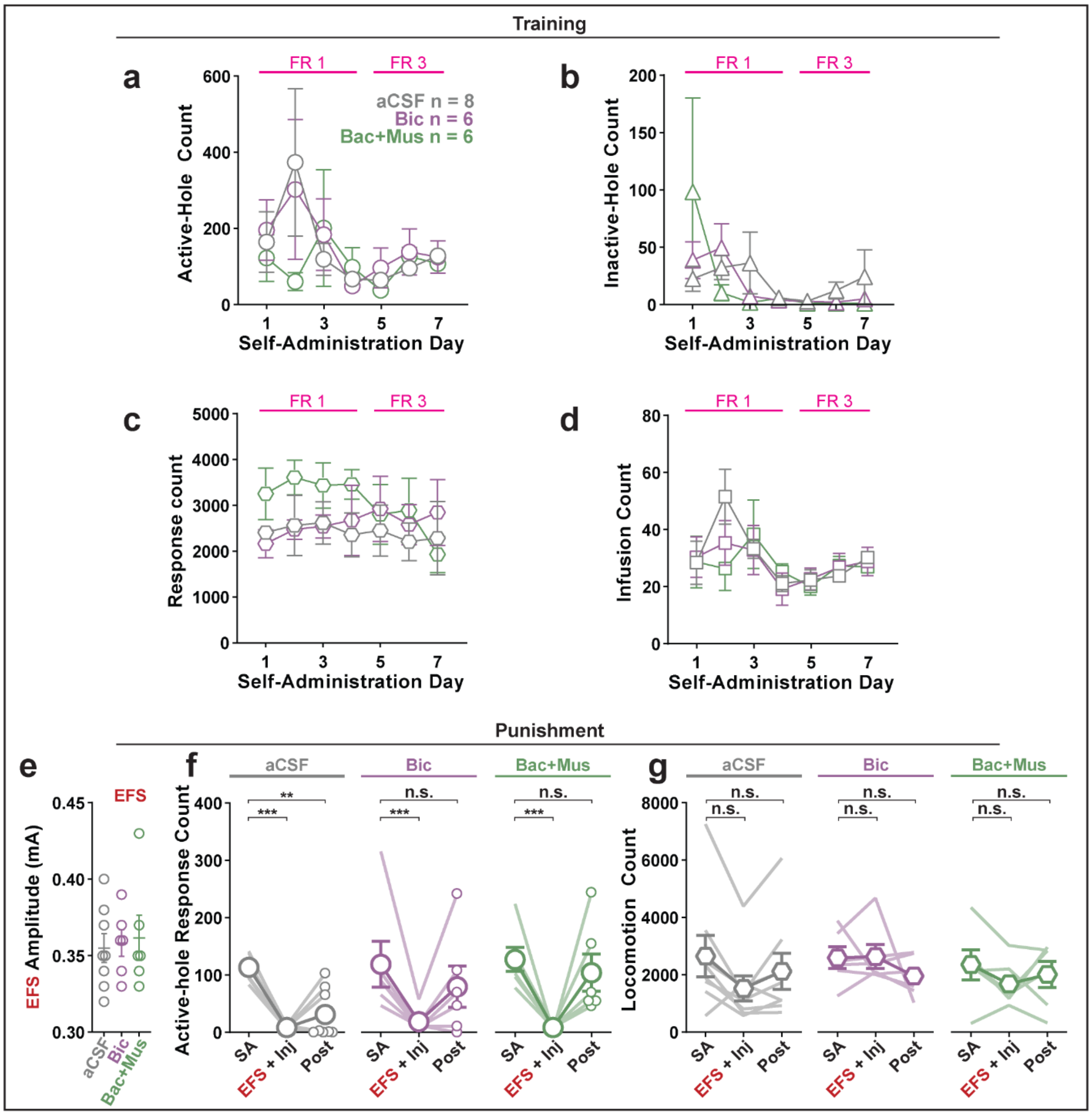

(a-d) Behavior during cocaine self-administration (SA); FR: fixed ratio (number of responses required to obtain one cocaine infusion). **(a)** active-hole responding, **(b)** inactive-hole responding, **(c)** infusions, and **(d)** locomotion. **(e)** Amplitudes of electric foot-shock (mA) given to each rat and group averages. **(f-g)** Behavior before punishment (SA), during punishment (EFS + Inj) and after punishment (Post). **(f)** Active hole responding. Punishment suppressed responding in all groups; after punishment, only animals microinjected with aCSF remained below baseline responding whereas those receiving bicuculline or baclofen + muscimol returned to pre-punishment levels (HSD, *** $P < 0.001$, ** $P < 0.01$). **(g)** Locomotion: there were no effects of microinjections or punishment on locomotion. For **a-d,** symbols are mean ± SEM for each group; for e Symbols are mean ± SEM for each group; lines and small points are individual subjects.

Supplementary figure 6: Subject Removals

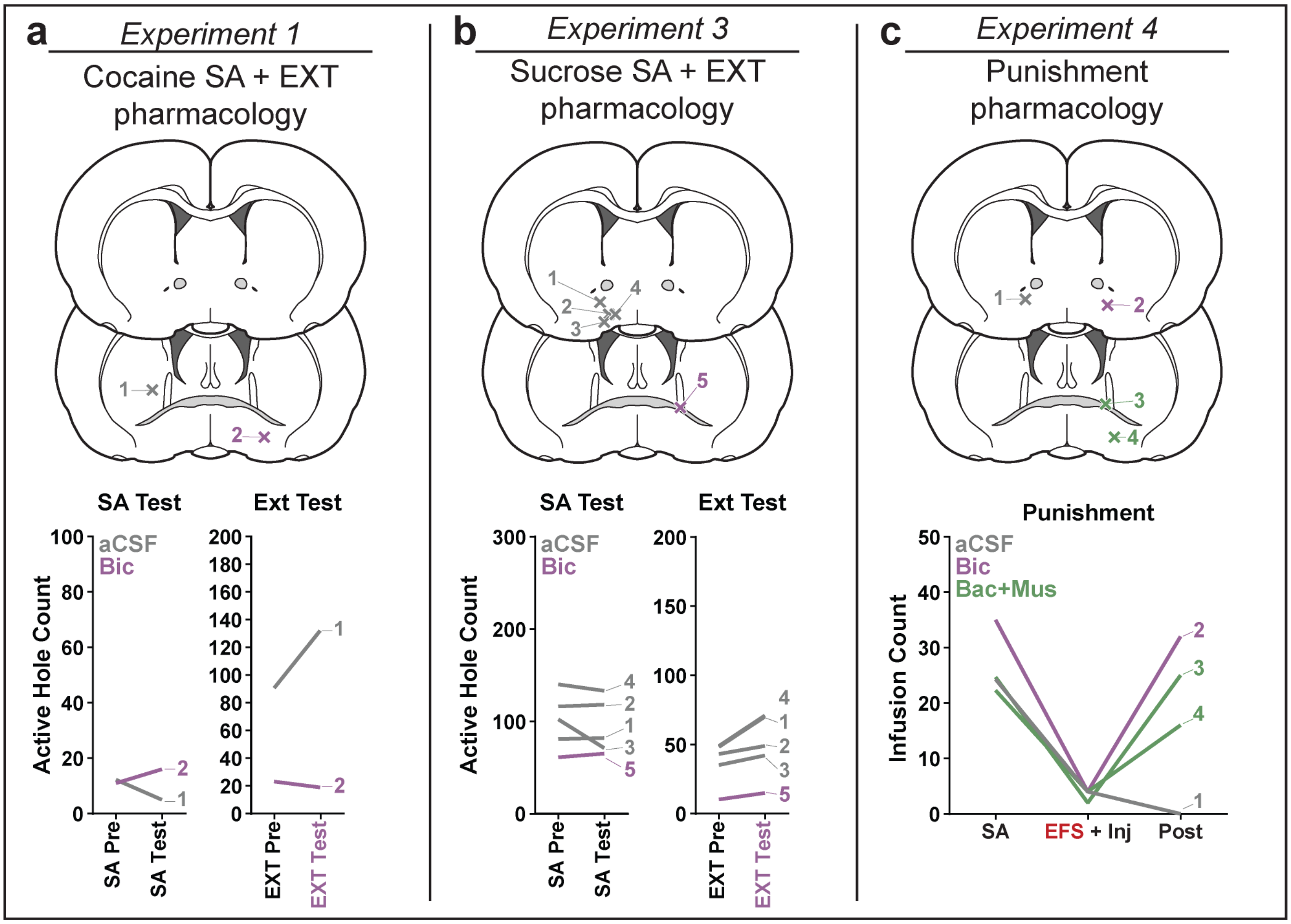

(a-c) position of subjects removed for misplacement of the cannulas (top) and the critical behavior relevant to each experiment (bottom). Color indicates corresponding intra_LPO injection: aCSF (grey), bicuculline (Bic, purple), and baclofen + muscimol (Bac+Mus, green). Number labels on bottom indicate the position of the corresponding fiber on top.
